# First Reported Case of Hyperchloremic Non-Anion Gap Metabolic Acidosis in a Patient Undergoing Continuous Bladder Irrigation for Hemorrhagic Cystitis

**DOI:** 10.7759/cureus.12132

**Published:** 2020-12-17

**Authors:** Azka Latif, Joseph Thirumalareddy, Akshat Sood, Sunil Nair, Abubakar Tauseef

**Affiliations:** 1 Internal Medicine, CHI Creighton University, Omaha, USA; 2 Hospital Medicine, Creighton University School of Medicine, St. Joseph's Hospital and Medical Center, Omaha, USA; 3 Internal Medicine, Creighton University School of Medicine, Omaha, USA; 4 Internal Medicine, Creighton University, Omaha, USA

**Keywords:** hyperchloremic, non-anion gap metabolic acidosis, undergoing, bladder irrigation, cystitis

## Abstract

Radiation cystitis can present as gross hematuria and occurs secondary to irritation of the bladder urothelium. Continuous bladder irrigation (CBI) is commonly used for the treatment of hemorrhagic cystitis for evacuation of blood clots and to maintain catheter drainage. Most commonly, CBI is performed using 0.9% sodium chloride. We report a 77-year-old female who developed hyperchloremic non-anion gap metabolic acidosis (H-NAGMA) and pulmonary edema secondary to absorption of 0.9% normal saline (NS) from CBI. In such cases, ringer lactate with low concentration (109 mEq) of chloride as compared to NS (154 mEq) can prove to be a suitable alternative.

## Introduction

In patients who have undergone radiation for the treatment of various pelvic malignancies or conditions, there is a possible side effect of radiation-induced hemorrhagic cystitis. Radiation cystitis can present in either an acute or chronic fashion. Late radiation cystitis often presents as gross hematuria and occurs secondary to irritation of the bladder urothelium. Continuous bladder irrigation (CBI) is commonly used for the treatment of hemorrhagic cystitis for evacuation of blood clots and to maintain catheter drainage. Most commonly, CBI is performed using 0.9% sodium chloride. The urothelium acts as a permeability barrier between urine and blood [1]. One of the main features of bladder includes low diffusive permeability to water [2]. Disruption of this barrier function in certain circumstances such as infections, radiations, toxic chemicals, and mechanical damage leads to reabsorption of fluid and solutes in the blood stream. Herein, we report a 77-year-old female who developed hyperchloremic non-anion gap metabolic acidosis (H- NAGMA) and pulmonary edema secondary to absorption of 0.9% normal saline (NS) from CBI.

## Case presentation

A 77-year-old female presented to the ED with a chief complaint of hematuria for one day. The patient had a past medical history of uterine cancer, 10 years prior, for which she received radiation in addition to a hysterectomy with bilateral oophorectomy. The recurrent hematuria and clots in the bladder were previously deemed to be secondary to hemorrhagic radiation cystitis from the previous radiation to the pelvis. During the course of stay, the patient required CBI for removal of clots of up to 12-15 L within the bladder and to prevent urinary retention. On hospital day 3, it was noted that the patient’s catheter was no longer draining. Bladder scan showed 500 mL at this time, and irrigation was attempted with another 780 mL of NS, after which several clots were noted to pass. Eventually the patient required frequent manual irrigation in order to maintain patency of the Foley catheter. By hospital day 4, the patient developed further abdominal distension and increased work of breathing including desaturation down to 80%. Chest X-ray was suggestive of fluid overload. Ultrasound abdomen showed no signs of hydronephrosis. Upon reinsertion of a new catheter, 10 L of fluid was drained, and the patient’s shortness of breath began to remit after treatment with diuretics. Daily laboratory testing showed low serum bicarbonate level at 14.0 mmol/L, albumin was 4.5 g/dL with a concomitant increase in serum chloride to 118 mmol/L (Tables 1-2).

**Table 1:** Basic metabolic profile during hospital stay.

Days after admission	Day 1	Day 2	Day 3	Day 3	Day 4	Day 5	Day 6	Day 7	Day 8	Day 9	Day 10	Day 11	Day 12	Day 13	Day 15	Day 18
Sodium	137	138	138	136	136	140	143	147	146	143	142	144	147	144	142	140
Potassium	4.7	5.0	4.7	4.7	4.3	3.9	3.8	3.4	3.8	3.2	3.8	3.7	4.0	3..6	3.5	4.2
Chloride	105	107	107	108	107	117	118	122	122	117	119	121	121	118	113	110
CO2	21.0	22.0	21.0	18.0	21.0	16.0	14.0	12.0	11.0	14.0	13.0	14.0	15.0	17.0	19.0	22.0
Anion gap	16	14	15	15	12	11	15	16	17	15	14	13	15	13	14	12
Calcium	9.0	8.3	8.2	8.2	8.1	6.6	6.3	6.3	6.0	6.3	7.2	7.4	7.1	7.5	7.5	7.2

**Table 2:** Urine electrolytes studies done prior to CBI. CBI, continuous bladder irrigation

Chloride, urine	132 (H)
Creatinine urine random	15 (L)
Osmolality random urine	306
Potassium random urine	5.7 (L)
Sodium, urine	135 (H)

Our differential diagnosis included renal tubular acidosis and H-NAGMA secondary to diarrhea. As there was no recent history of diarrhea, it was excluded. To rule out renal tubular acidosis urine studies were performed. Nephrology was consulted, and she was started on 650 mg of sodium bicarbonate three times daily along with a hypotonic bicarbonate infusion according to the guidelines, in order to improve the bicarbonate. Over the course of next few days, the rate of CBI was weaned down, with as needed intermittent manual irrigation. Acidosis was not improving after bicarbonate tablets, but as the CBI was weaned acidosis resolved with improvement of serum bicarbonate (22 mmol/L) on day 18.

## Discussion

Most common etiologies of hemorrhagic cystitis are infections, anticancer chemotherapy, and pelvic radiation. Radiation cystitis is a late complication and it can occur as late as after 10 years of radiation therapy [3], as it was seen in our patient 10 years post radiotherapy. The treatment of radiation cystitis includes CBI for the evacuation of blood, use of hyperbaric oxygen to promote healing of urothelium [4], and surgical options such as cystoscopy, fulguration, and nephrostomy tube insertion.

The CBI with 0.9% NS is the standard of care for clot evacuation. Elliot et al. studied exfoliation rates of urothelial cells in patients (N=5) with chronic urinary tract infections and in patients with long-term indwelling catheters. They observed bladder irrigation was associated with an increased disruption of urothelial cells which in turn predisposes the bladder to recurrent infections [5]. In 2014 Paolo et al. reported acute severe pulmonary edema in an 85-year-old male who underwent CBI. Autopsy performed on bladder sample revealed a highly damaged urothelium with mucosal and interstitial edema on histological and immunohistochemical study [6]. Likewise, in our patient bladder irrigation led to systemic absorption of fluid from bladder urothelium that manifested as NAGMA. In our patient, as the serum chloride level started to increase, it decreased serum HCO3 causing H-NAGMA. Once we held bladder irrigation, the chloride levels decreased followed by improvement of HCO3 and resolution of acidosis. As the patient was on diuretic therapy for pulmonary edema urine studies were considered unreliable. NS has 154 mEq of chloride, and fluid resuscitation with NS leads to an overload of chloride ions into the blood, subsequently leading to H-NAGMA. Chloride and bicarbonate ions work together to maintain an ionic balance of the cellular space. Hyperchlorhydria favors intracellular transfer of bicarbonate ion to maintain ionic equilibrium, thus reducing its availability to the pH buffer system ultimately causing net acidosis [7]. As, the patient was not on IV fluids suggesting that systemic absorption (approximately 10L) occurred from bladder urothelium leading to pulmonary edema and acidosis.

Our case demonstrates that CBI can cause massive absorption of fluids from bladder epithelium causing NAGMA and pulmonary edema. In such cases, ringer lactate with low concentration (109 mEq) of chloride as compared to NS (154 mEq) can prove to be a good alternative.

## Conclusions

Continuous bladder irrigation is associated with an increased disruption of urothelial cells which in turn predisposes the bladder to recurrent infections. CBI with 0.9% NS is the standard of care for clot evacuation. Continuous bladder irrigation may lead to systemic absorption of fluid from bladder urothelium that can manifest as NAGMA. In such cases, ringer lactate with low concentration (109 mEq) of chloride as compared to NS (154 mEq) can prove to be a good alternative.
